# Optimization of a novel large field of view distortion phantom for MR‐only treatment planning

**DOI:** 10.1002/acm2.12090

**Published:** 2017-05-12

**Authors:** Ryan G. Price, Robert A. Knight, Ken‐Pin Hwang, Ersin Bayram, Siamak P. Nejad‐Davarani, Carri K. Glide‐Hurst

**Affiliations:** ^1^ Department of Radiation Oncology Henry Ford Health System Detroit MI USA; ^2^ Department of Neurology NMR Laboratory Henry Ford Health System Detroit MI USA; ^3^ Department of Radiation Oncology Wayne State University School of Medicine Detroit MI USA; ^4^ Department of Imaging Physics University of Texas MD Anderson Cancer Center Houston TX USA; ^5^ MR Applications & Workflow GE Healthcare Houston TX USA

**Keywords:** distortion, gradient nonlinearity, MRI, phantom, spatial accuracy

## Abstract

**Purpose:**

MR‐only treatment planning requires images of high geometric fidelity, particularly for large fields of view (FOV). However, the availability of large FOV distortion phantoms with analysis software is currently limited. This work sought to optimize a modular distortion phantom to accommodate multiple bore configurations and implement distortion characterization in a widely implementable solution.

**Method and Materials:**

To determine candidate materials, 1.0 T MR and CT images were acquired of twelve urethane foam samples of various densities and strengths. Samples were precision‐machined to accommodate 6 mm diameter paintballs used as landmarks. Final material candidates were selected by balancing strength, machinability, weight, and cost. Bore sizes and minimum aperture width resulting from couch position were tabulated from the literature (14 systems, 5 vendors). Bore geometry and couch position were simulated using MATLAB to generate machine‐specific models to optimize the phantom build. Previously developed software for distortion characterization was modified for several magnet geometries (1.0 T, 1.5 T, 3.0 T), compared against previously published 1.0 T results, and integrated into the 3D Slicer application platform.

**Results:**

All foam samples provided sufficient MR image contrast with paintball landmarks. Urethane foam (compressive strength ∼1000 psi, density ~20 lb/ft^3^) was selected for its accurate machinability and weight characteristics. For smaller bores, a phantom version with the following parameters was used: 15 foam plates, 55 × 55 × 37.5 cm^3^ (L×W×H), 5,082 landmarks, and weight ~30 kg. To accommodate > 70 cm wide bores, an extended build used 20 plates spanning 55 × 55 × 50 cm^3^ with 7,497 landmarks and weight ~44 kg. Distortion characterization software was implemented as an external module into 3D Slicer's plugin framework and results agreed with the literature.

**Conclusion:**

The design and implementation of a modular, extendable distortion phantom was optimized for several bore configurations. The phantom and analysis software will be available for multi‐institutional collaborations and cross‐validation trials to support MR‐only planning.

## 1 Introduction

Due to the superior soft tissue contrast provided by magnetic resonance imaging (MRI), its use can provide increased delineation accuracy over computed tomography (CT) for radiation treatment planning^1^, ^2^. However, implementation of MRI into treatment planning may be limited by both system‐level and patient‐induced geometric distortions^3^, ^4^. The magnitude of patient‐induced distortions arise from susceptibility differences within the patient and chemical shift effects, while system‐level distortion is a result of B_0_ field inhomogeneity and gradient nonlinearity (GNL). While patient‐specific distortion is dependent on field strength and acquisition parameters and thus must be minimized on a per‐scan basis, GNL‐induced distortions have been shown to be independent of acquisition sequence^5^. As one of the dominant sources of image distortion^6^, GNL distortion is further exacerbated by modern systems with fast slew rates^7^ or by systems with an ‘open’‐bore design.^8^ These system‐specific distortions have been shown to increase with increased distance from isocenter, making accurate measurement and correction over large fields of view (FOVs) important for radiation treatment planning involving anatomy positioned away from isocenter.^8^

To characterize large FOV GNL distortion, several investigators have designed and constructed in‐house phantoms. Early designs include Tanner et al.*,* who utilized orthogonal arrays of water‐filled polymethyl methacrylate (PMMA) tubes to characterize a volume of 40 × 25 × 40 cm^3^ (in the left‐right (L‐R), anterior‐posterior (A‐P), and superior‐inferior (S‐I) axes, respectively)^9^. While the PMMA tubes have small susceptibility differences from water, they also expanded/contracted substantially with temperature changes, and necessitated the use of free‐sliding seals at tube support positions. Breeuwer et al. used a 3D array of point‐like landmarks^10^ while Wang et al. used a 3D grid spanning a 31 × 31 × 31 cm^3^ volume^3^. Both of these phantoms required a fluid filling to serve as contrast from the markers. More recently, Huang et al. devised a hybrid design comprised of regularly spaced spherical cavities connected by channels in a grid‐like pattern^11^. This design also utilized liquid contrast filling, but unlike the others, directed the contrast into the hollow landmarks themselves, creating the potential for air bubbles. Also, while large in the axial plane (46.5 × 35 cm^2^), they did not provide full S‐I FOV characterization, spanning a distance of only 16.8 cm in that dimension. Walker et al. developed a full FOV distortion phantom, utilizing an array of vitamin E capsules over a diameter of 500 mm and length of 513 mm and used this phantom to characterize the entire FOV for a 3T Siemens system^12^.

While many in‐house 3D distortion phantoms have been developed, some of the current designs are limited by a single geometric configuration to accommodate the institution's particular MRI system. While Walker et al.'s phantom configuration was modular, this was not explored in their recent publication^12^. Furthermore, although various phantoms have been created, the availability of comprehensive distortion analysis software is currently limited. Thus, the goal of this work was to evaluate the phantom design needs of the MR‐SIM community based on currently available platforms and bore sizes and to develop a modular large FOV phantom using easily obtainable materials that can be optimized for many MR systems. Lastly, in‐house distortion characterization software was optimized for several MR platforms and integrated into a widely available medical imaging application platform. Importantly, the modular phantom design and availability of standardized analysis can be used in the future to facilitate collaboration and perform benchmarking for multi‐institutional trials of MR‐only treatment planning.

## 2 Materials and Methods

### 2.A Phantom materials

The phantom design utilized in this work was adapted from a previously described study^13^ that used a stack of low‐density polyurethane foam plates (6 lbs/ft^3^, 2.5 cm thick) with 6 mm paintball inserts (polyethylene base) as signal generators (available at: http://www.MCSUS.com, UPC: 844596050069). While the original phantom design was lightweight, the low‐density foam was found to be pliable and easily damaged, making long‐term stability of the phantom's geometric integrity a potential concern. To build a more robust phantom with a material that could withstand transport to multiple Radiation Oncology centers for benchmarking, twelve urethane foam‐based materials of various density and strength characteristics (4–40 lbs/ft^3^ and 8–72 Shore D hardness, where Shore D is a hardness scale commonly used for plastics and elastomers^14^) were identified. Test slabs were custom machined by Non‐Magnetic Specialties for each candidate material (25 ± 0.25 mm center‐to‐center spacing, ~6.5 mm deep using a ~6.4 mm ball nosed endmill) and 6 mm paintballs were inserted into the foam. MR and CT images were acquired to assess the paintball signal intensity relative to each background material. Because CT will serve as the “ground truth” image for distortion calculations, intensity‐based automatic segmentation of the paintballs from the background material was an important consideration. Final material selection was performed based on a balance of strength, weight, machinability, and cost.

**Figure 1:**
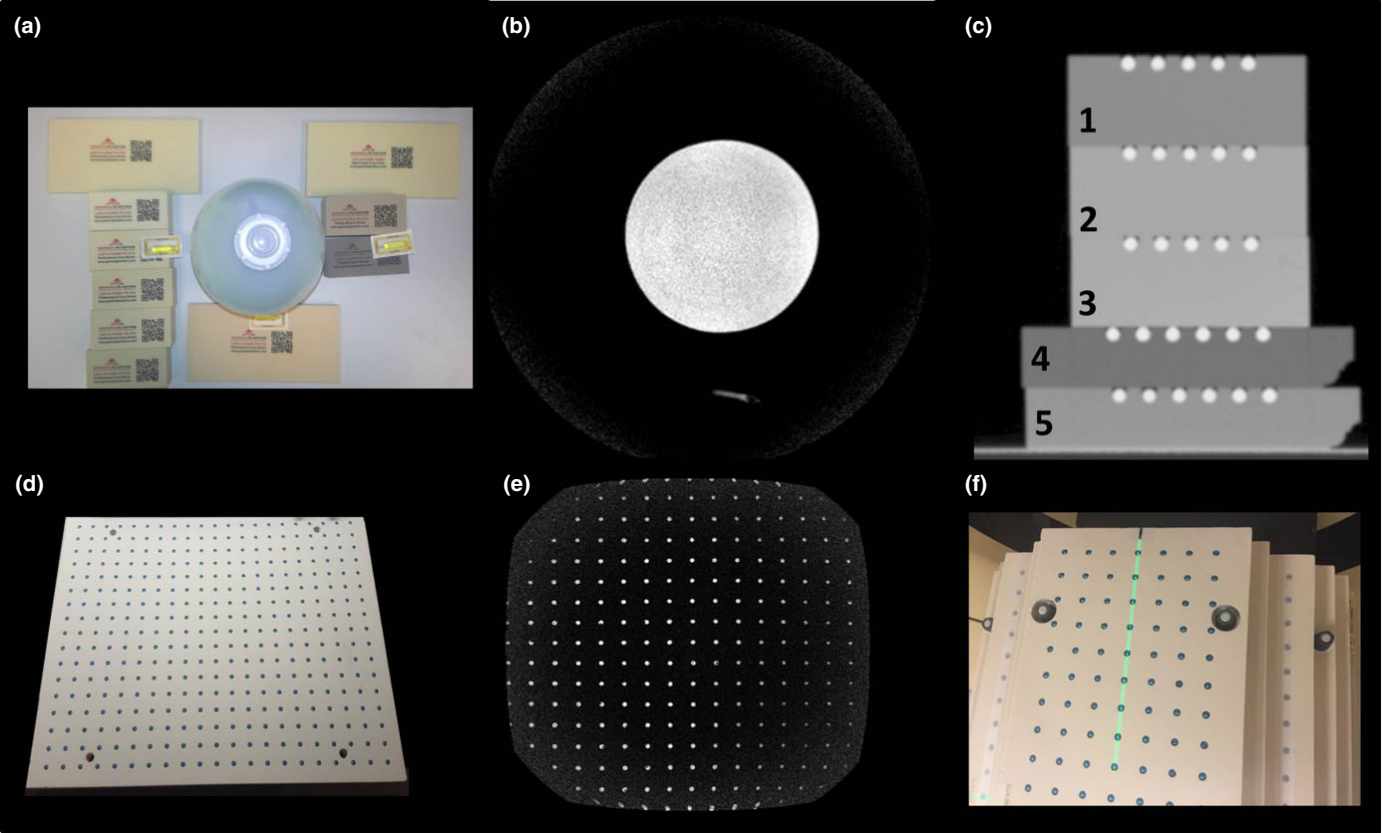
a, Polyurethane foam samples that were evaluated for MRI and CT signal studies. The signal generator bottle in the center was used for reference. b, Axial cross‐section of a 1.0T T1‐weighted image illustrating the lack of signal from the polyurethane materials. c, Coronal CT image of five selected polyurethane plates that were precision‐machined and fitted with paintballs used for the signal analysis study with phantom densities ranging from 20 to 40 lbs/ft^3^ and were found to have acceptable machining characteristics. d, Example of a finalized precision‐machined plate illustrating the paintball landmarks and fiberglass threaded rods in the corners of the plate to improve stability. e, Coronal slice 1.0T MR image of completed plate. f, Anterior view of the assembled 3D distortion phantom highlighting the high‐strength fiberglass threaded rods used to assemble the phantom and improve stability.

Eight high‐strength fiberglass threaded rods (McMaster‐Carr, Part #91315A231) with corresponding nuts were used to affix the phantom plates together (four placed in the corners of the largest plates and an additional four that affixed the smaller plates to the largest ones) and add stability to the phantom construction as shown in Fig. 1F. The dimensions of the rod holes were machined with a tolerance of ±0.125 mm. Once the plates were aligned in the stack, the nuts were tightened to add additional stability to the phantom assembly.

### 2.B Bore/phantom model

Bore sizes and minimum aperture widths (smallest diameter of clearance within the bore once the couch is positioned inside) were tabulated for fourteen MR systems and one MR‐IGRT system across five vendors as shown in Table 1. An in‐house MATLAB^®^ (Mathworks, Natick, MA, USA) script was used to generate shape models of each bore geometry, with input constraints including (1) the physical bore sizes and (2) the minimum aperture widths (smallest diameter of clearance within the bore once the couch is positioned inside) for each MRI make/model, assuming a flat table top was used. Optimized phantom configurations for each bore model were then generated by iteratively varying the phantom slab widths and total number of slabs until an optimized geometrical phantom configuration was found using the largest FOV physically possible. In order to simplify the model, the script assumes a circular cross‐sectioned bore for all MR systems other than the Philips Panorama High Field Open (HFO) and a flat couch‐top. Nonetheless, it was useful for visualization and planning of the final phantom construction.

**Table 1:** Bore sizes, FOV, and minimum aperture widths resulting from couch position tabulated for fourteen MR and one MR‐IGRT systems across five vendors

MR system vendor	Model	Bore size (cm)	Min. aperture (cm)	FOV (cm^3^)
GE	Signa (1.5 T)	60	46.5	48 × 48 × 48
	Optima MR450w	70	52	50 × 50 × 50
	Discovery MR750w			
Philips	Intera	60	42	53 × 53 × 53
	Panorama	Open	45	45 × 45 × 45
	Achieva	60	42	53 × 53 × 53
	Ingenia	70	53	55 × 55 × 50
Siemens	Symphony	60	45.2	50 × 50 × 50
	Avanto		45.5	
	Aera	70	55	
	Skyra			
	Verio			
Toshiba	Vantage	60	48.3	50 × 50 × 50
	Titan	69	52.9	55 × 55 × 50
ViewRay	MRIdian	70	55	50 × 50 × 50

### 2.C Phantom setup reproducibility

To evaluate phantom setup reproducibility, 5 repeat CTs with independent setup and alignments to the CT external lasers were performed. DICOM CT data of Trials 2–5 were rigidly registered to Trial 1 using the previously validated FMRIB's Linear Image Registration Tool (FLIRT) module in the FMRIB Software Library (FSL)^15^, ^16^. Six parameter (translation and rotation) and three parameter (translation only) rigid registrations were performed using the spline function for interpolation and mutual information as the cost function.

### 2.D Software design

In‐house image processing software was developed in C++ to automatically generate geometric distortion maps from phantom DICOM MRI data using similar techniques described in detail in our previous work^8^ assuming the reverse gradient methodology is used (described in detail in Section 2.E). The useful marker signal was extracted from the image using a connectivity algorithm combined with masking and thresholding. Finally, x, y, and z control point positions were determined by finding the centroid of each marker as described in a previous publication^8^. The central control point is then identified on both the MR and CT image, and combined with DICOM header information to perform a coordinate transformation of the CT control point positions to the MR coordinate space. Total distortion at each control point was then calculated by measuring the difference between MR control point positions with those generated from the reference CT image for that particular phantom configuration. Full distortion maps were then generated across the entire FOV by interpolation using singular value decomposition to fit the data to a sixth‐degree polynomial as previously implemented^17^.

To make our work widely available to the community, we integrated our distortion characterization software into the 3D Slicer application platform^18^. 3D Slicer is an extensive medical image processing toolset, widely available open‐source code, and modular design that is designed as a plugin framework. This then allowed for our distortion software to be written as a loadable C++ module that can utilize any of the robust C++ libraries already integrated into the 3D Slicer core. Specifically, our module uses existing DICOM import plugins, as well as existing VTK^19^ visualization mechanisms, Qt^20^ for user‐interface construction, and both ITK^21^ and VTK for image processing. C++ also offers the advantage of faster run‐times as compared to MATLAB and other computing software.

### 2.E Software evaluation

To evaluate the 3D Slicer software performance, GNL was evaluated for the 1.0 T HFO MR‐SIM and compared against our previously published results using MATLAB and a different large FOV distortion phantom as described by Huang et al.^11^. Our previous work illustrated that the GNL for this magnet was stable compared to baseline measurements over more than 6 months of operation, thus suggesting that benchmarking with this magnet was appropriate. Distortion maps were compared directly via difference maps within the FOV covered by both phantoms. Global distortion statistics (including the percent of voxels distorted over 1, 2, 3, 4, and 5 mm and maximum distortions) were also compared between approaches, and comparisons in polynomial data fits were evaluated based on the mean absolute error. Finally, distortion maps were plotted as a function of radial distance from isocenter to compare the overall distribution of new distortions maps with those that we were previously validated.

It is important to note that exact agreement cannot be expected between the previously measured data using a different phantom and software and our new modular phantom. While the model fitting (singular value decomposition to fit the data to a sixth‐degree polynomial, magnet measured, and acquisition sequences) were identical between trials, major differences between the approaches include: different reference sets (our previous version used a computerized binary template while the new one uses a CT reference scan with 2 mm slice thickness), static measurement (single couch position for our large FOV phantom) compared to the stepped couch required to accommodate the old phantom's smaller SI extent of 16.8 cm, and the overall number and resolution of the control points (4,600 spaced 16 mm apart and up to ~7,500 spaced 25 mm apart for the old phantom and new phantom, respectively). Nevertheless, it is important to benchmark the new results against previously validated and published data.

### 2.F Multiple magnet distortion characterization

CT reference images were acquired of the phantom in each configuration using a large‐bore multislice CT scanner (Brilliance™ CT Big Bore v3.6; Philips Healthcare, Cleveland, OH, USA) at 120 kVp, 344 mAs, and voxel dimensions 1 × 1 × 2 mm^3^. MR images were acquired on three MR systems: a 1.0 T Panorama High‐Field Open 45 cm bore, version 3.5.2), 1.5 T 60 cm wide bore Ingenia (version 4.1.3), and a 3.0 T Ingenia with a 70 cm wide bore (version 5.7.7, Philips Medical Systems, Cleveland, OH, USA). All images were acquired using integrated quadrature coils with a 3D T1‐weighted gradient‐echo sequence with acquisition parameters shown in Table 2. Note that despite the bore geometry being different between magnets tested (i.e., vertical vs horizontal configurations), the reported results are in the head‐first supine patient orientation.

**Table 2:** MRI acquisition parameters for each of the three MR systems tested in the multimagnet characterization study

	1.0 T Panorama	1.5 T Ingenia	3.0 T Ingenia
Bore geometry	Vertical	Horizontal	Horizontal
TE(ms)	5.5	4.4	2.98
TR(ms)	30	30	31.74
Flip angle(°)	28	28	28
Acquisition matrix	432/430	432/433	296/297
Bandwidth (Hz/pixel)	190	190	433
Reconstructed voxel size (mm^3^)	0.96 × 0.96 × 2	0.77 × 0.77 × 2	0.61 × 0.61 × 2
Signal averages	1	1	1
# Phantom slabs	15	15	17
# Useable landmarks	5,082	5,082	6,048

Two scans were obtained for each MRI acquisition with fixed parameters except for using a forward or reverse read gradient polarity. In this manner, the GNL‐induced distortion could be isolated from total distortion using the reverse gradient methodology,^6^, ^22^, ^23^, ^24^ and allowed for generation of distortion correction maps. Standard 3D gradient echo imaging protocols utilize phase encoding for two axes with only one frequency encoded axis, which isolates object‐dependent and B_0_‐related distortions to this axis, as they are only present in frequency encoding directions. Distortions resulting from GNL are present in all directions, and are independent of acquisition sequence. Also, when the polarity of the read gradient is reversed, the polarity of any B_0_ distortions will also be reversed while GNL distortion remains constant, and thus, the GNL distortion can be isolated by taking the average distortion between the two scans.

All scans were acquired with vendor supplied 3D geometry corrections enabled. Thus, it is important to note that all data shown are after vendor corrections were applied and thus represent the residual distortion in the datasets. The corresponding MR and CT scans for three phantom configurations were then uploaded into 3D Slicer for GNL and distortion analysis. Also, as each MR system produced images of different contrast, resolution, and signal to noise, the parameters utilized for thresholding and object identification were changed for each magnet to yield optimal results.

## 3 Results

### 3.A Final phantom design and construction

Figure 1 shows the setup and corresponding MR images for the initial signal test as well as CT images of the polyurethane foam plates used in the CT contrast analysis. All urethane foam materials did not provide measurable MR signal and were thus considered adequate for our purposes. Materials with densities less than 20 lbs/ft^3^ were found to be too brittle for precise machining; the materials were prone to crumbling and did not hold their precision‐machined shapes. Thus, signal analysis was performed on the five foam samples that met the ≥20 lbs/ft^3^ criteria. CT signal was found to be −547, −396, −382, −680, and −505 HU for the materials shown in Fig. 1 (numbered 1–5) respectively. The contrast between the foam layer and corresponding paintballs embedded in that particular slab were 636, 483, 478, 769, and 592 HU for materials 1–5, respectively. Thus, in order to achieve optimal contrast from the paintballs and maintain the lowest reasonably achievable weight without sacrificing machinability, the 20 lbs/ft^3^ material (Coastal Enterprises, Precision Board Plus High Density Urethane) shown in Fig. 1C, material #4 (−680 HU) was used for the final phantom construction. This final material was selected based on considerations of total phantom weight, strength, density, and machinability. The 20 lbs/ft^3^ plates were machined to 25 ± 0.5 mm thickness and the paintball holes were located in a 2‐D rectangular grid pattern (25 ± 0.25 mm center‐to‐center spacing, ~6.5 mm deep using a ~6.4 mm ball nosed endmill) for 6 mm diameter paintball marker placement.

**Figure 2:**
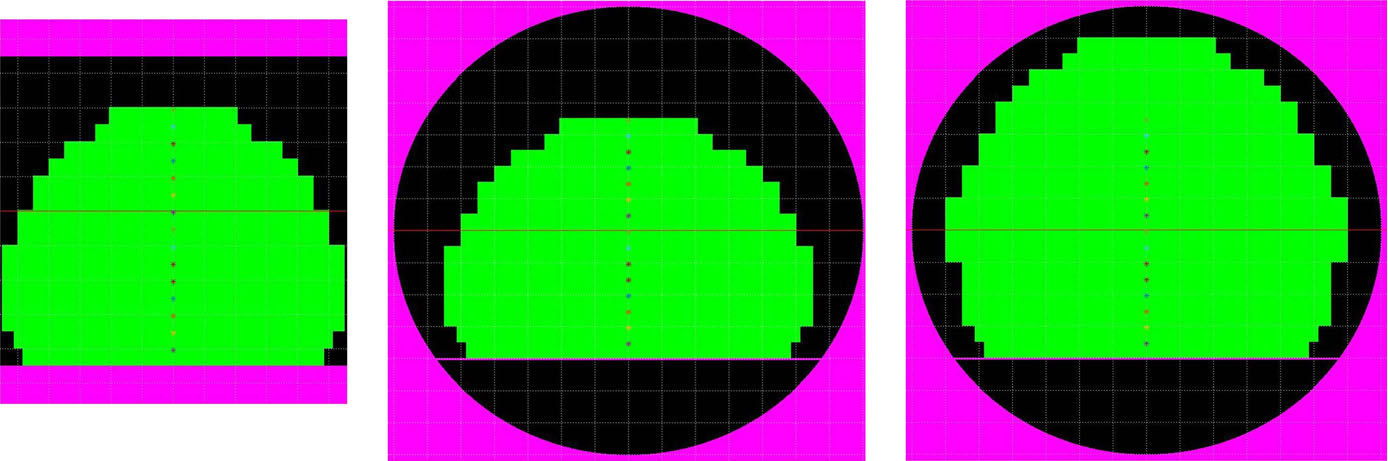
(Left) Open‐bore MRI with standard phantom construction (15 plates). (Middle) 60 cm bore magnet with standard phantom construction (15 plates). (Right) 70 cm bore magnet with extended phantom construction (20 plates).

Figure 2 depicts various modeled bore and phantom arrangements as simulated by MATLAB. The left side shows the open‐bore Philips Panorama, the middle shows the 60 cm cylindrical bore configurations, and the right shows a 70 cm bore configuration. The illustrated phantom design for the left and middle pane utilizes a stack of 15 plates (2.5 cm thick), and a FOV of 55 × 55 × 37.5 cm^3^ (L‐R, S‐I, AP), and while this design works well for the 60 cm bores, it leaves a significant portion of the FOV in the 70 cm bore uncharacterized. For this reason, we chose to build the phantom using a modular design with two main configurations: (1) the standard build as shown in Fig. 2, and (2) the extended build, which utilizes a stack of 20 plates and a final FOV of 57.5 × 55 × 50 cm (L‐R, S‐I, AP). The right panel of Fig. 2 is illustrates this extended build in a 70 cm bore.

Additional holes were drilled and fit with fiberglass tubing inserts to allow the plates to be stacked, with the plates held together using 3/8 inch diameter and 16 threads per inch fiberglass rods and hardware to secure the stack together once the paintballs were loaded. One advantage of using this modular design was that each successive plate in the stack locks the paintballs into the plate below it.

### 3.B Phantom setup reproducibility

The modular phantom setup was found to be very reproducible between different experiments; rigid registration with three parameters resulted in translations of 0.12 ± 0.04 mm, 0 ± 0 mm, and −0.61 ± 0.13 mm along the X, Y, and Z axes, respectively. Rotations were found to be negligible (~0°) when a six‐parameter (translation and rotation) method was used with stable translation results: 0.12 ± 0.02 mm, 0.001 ± 003 mm, and −0.35 ± 0.57 mm along the X, Y, and Z axes, respectively.

### 3.C Software design

**Figure 3:**
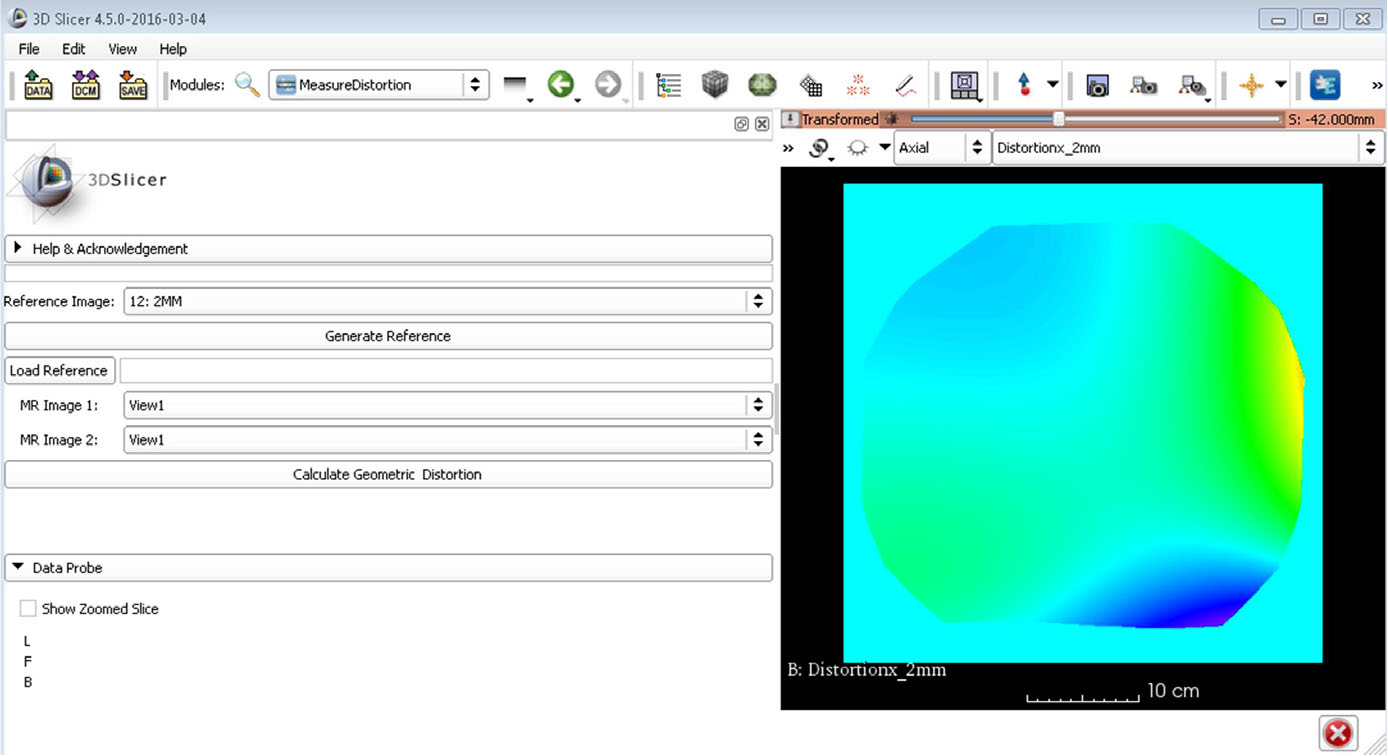
3D Slicer distortion module graphic user interface for 3D gradient nonlinear distortion assessment.

Figure 3 shows the graphic user interface developed for the Beta version of the distortion module integrated into 3D Slicer. Utilizing previously implemented tools and existing VTK, ITK, and Qt libraries, our distortion characterization software was integrated into the 3D Slicer tool set. Using C++ as the primary language of implementation, the total run‐time was approximately 8 min for an Intel Core i7‐4770 CPU. When compared to our previous MATLAB code for a similarly sized phantom, the overall run‐time efficiency gain was ~50% (17 min for MATLAB vs 8 min for 3D Slicer).

### 3.D Software validation

**Figure 4:**
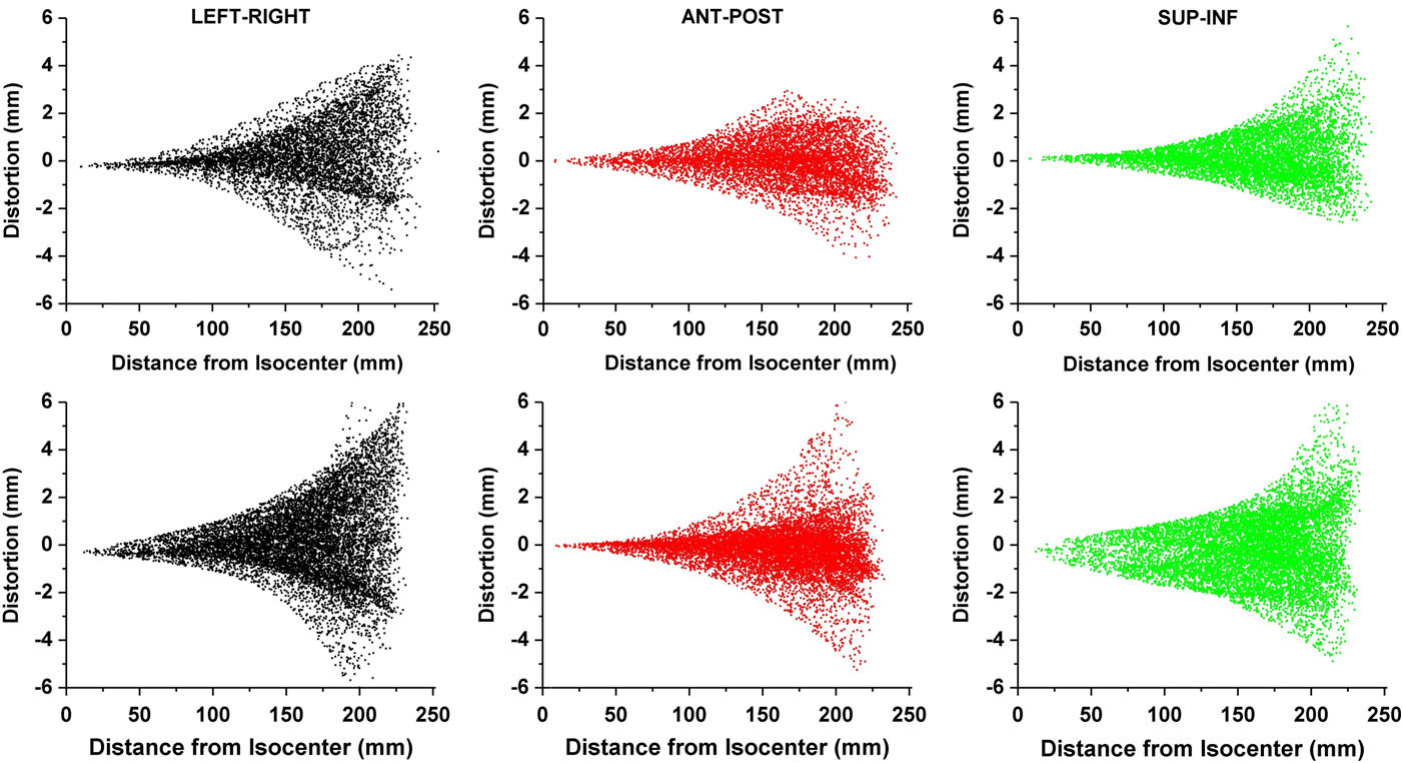
(Top Row) Distortion plotted as a function of radial distance from isocenter as generated with previously validated MATLAB software for the left‐right (LR), anterior‐posterior (AP), and superior‐inferior (SI) distortion from left to right, respectively. (Bottom Row) Similar distortion maps as measured with the new phantom and generated with 3D Slicer.

To evaluate the software performance, GNL was evaluated for our 1.0 T HFO MR‐SIM and compared against our previously published results. The plots shown in Fig. 4 demonstrate the distortion as a function of radial distance from isocenter in all three axes, where the top row was generated with the MATLAB software using a different 3D distortion phantom and the bottom row was generated using 3D Slicer and measured using the new modular distortion phantom. Both approaches measure similar distortion distributions, with the closest distortion greater than 1 mm occurring at ~10 cm for both the LR and AP axes. The greatest variation occurred in the SI direction, where the closest distortion > 1 mm occurred at ~10 cm for the approach utilizing the original phantom and MATLAB, but occurred closer to 5 cm for the approach using the modular phantom and 3D Slicer.

Table 3 summarizes the statistics for the measured GNL distortion and overall both the MATLAB/Phantom 1 (Method 1, established data) and 3D Slicer/Modular Phantom 2 GNL distortion measurements (Method 2, experimental data) revealed similar results in the A‐P and L‐R axes. However, the S‐I axis showed significantly more distortion for Method 2, with roughly 45% of voxels distorted more than 1 mm, while Method 1 measured about 25%. Nevertheless, the polynomial fit was found to be near equivalent for both methods, with mean absolute errors between the measured and modeled distortions of <0.1 mm different between methods.

**Table 3:** Comparison of gradient nonlinearity distortion statistics generated for 1.0 T Panorama to determine agreement between two approaches

	Established MATLAB/Phantom data (method 1)			Experimental 3D Slicer/modular phantom data (method 2)		
	L‐R	A‐P	S‐I	L‐R	A‐P	S‐I
Max distortion (mm)	5.5	4.2	6.1	8.2	6.5	8.7
Voxels distorted > 1 mm (%)	39.3	26.1	25.2	45.6	22.8	45.1
Voxels distorted > 2 mm (%)	14.8	3.2	5	20.0	5.9	12.8
Voxels distorted > 3 mm (%)	4.4	0.4	1.2	7.8	2.2	3.1
Voxels distorted > 4 mm (%)	0.5	<0.1	0.3	2.7	0.8	1.0
Voxels distorted > 5 mm (%)	<0.1	0	<0.1	0.7	0.2	0.3
Mean absolute error (mm)	0.3 ± 0.4	0.2 ± 0.2	0.5 ± 0.6	0.3 ± 0.4	0.3 ± 0.3	0.6 ± 0.6

### 3.E Multiple magnet distortion characterization

**Figure 5:**
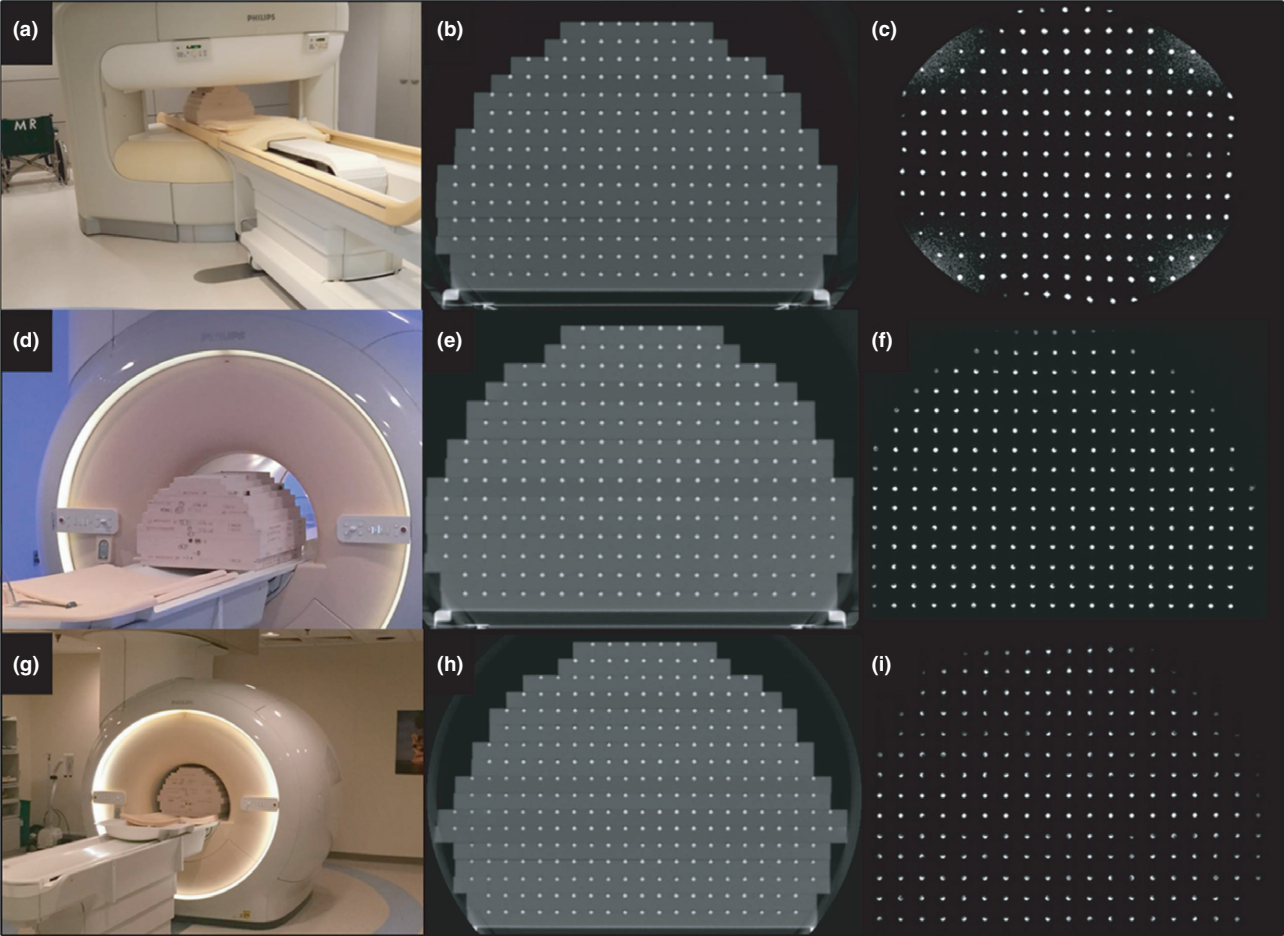
(a) Standard phantom configuration (15 plates) on the 1.0 T Philips Panorama with corresponding (b) CT image and (c) MR image. (d) Standard phantom experimental setup (15 plates) was also used for the 1.5 T Philips Ingenia with the corresponding CT (e) and MRI (f) shown. (g) Modified extended build (17 plates) scanned in the 3.0 T Philips Ingenia and the corresponding CT (h) and MRI (i) data.

Figure 5 illustrates the phantom setup and configuration for the three MRI units evaluated in this study. The standard build of 15 plates (FOV of 55 × 55 × 37.5 cm^3^) was used to characterize the 1.0 T Panorama (Fig. 5 A–C) and the 1.5 T Ingenia (Fig. 5 D–F). For the 3.0 T Ingenia wide bore, on the other hand, (Fig. 5 G‐I) an extended build of 17 plates (FOV of 55 × 55 × 45 cm^3^) was used. This deviated from the simulated extended FOV phantom build by three plates (initially planned to 50 cm height) due to clearance within the bore, although this also highlighted the importance of the modular design to accommodate the different architecture of each bore and couch combination.

**Figure 6:**
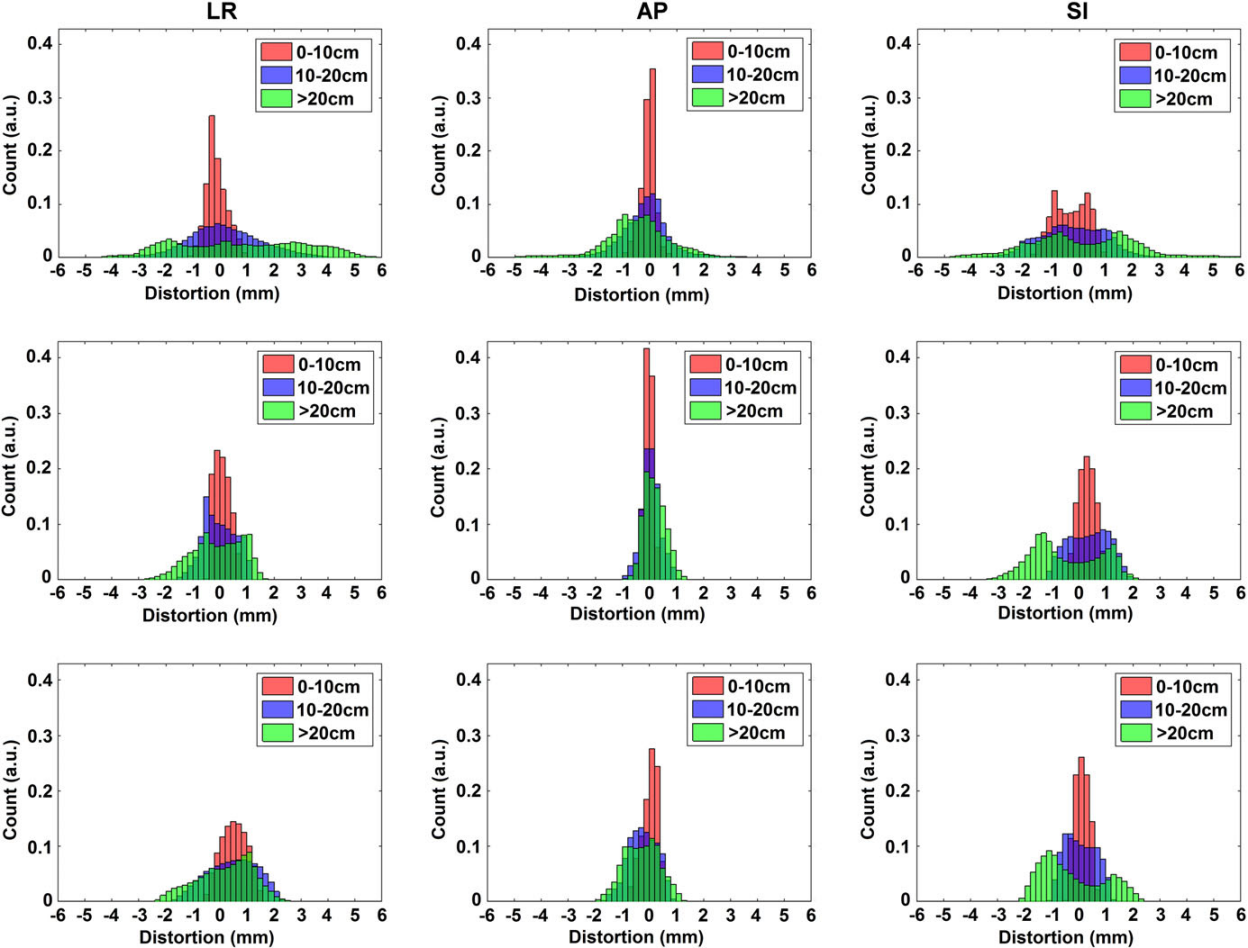
Histograms representing the distributions of distortion measurements for the left‐right (LR), anterior‐posterior (AP), and superior‐inferior (SI) directions using distance to isocenter groupings. Data are shown as follows: (Top Row) 1.0 T Panorama, (Middle Row) 1.5 T Ingenia, and (Bottom Row) 3.0T Ingenia Wide Bore.

Figure 6 summarizes the characterized GNL distortion distribution for the three MRI systems using data generated from 3D Slicer, and grouped into three radial distances from isocenter (0–10 cm, 10–20 cm, and > 20 cm). In general, both cylindrical bore systems revealed less GNL distortion than the 1.0 T Panorama although it is important to note that distortions > 1 mm do exist at FOV larger than 10–15 cm. All systems had less than 1 mm of distortion for radii less than 100 mm from the magnet isocenter, and started to deviate at distances above this for both the LR and SI directions. However, for the AP axis, both cylindrical bore systems nearly maintained less than 1 mm of distortion for the entire FOV.

While the 1.0 T Panorama yielded more than 1 mm of distortion in the L‐R direction for over 45% of voxels, the 1.5 T Ingenia yielded this magnitude of distortion for about 21% of voxels, and the 3.0 T for roughly 39% of voxels. Both cylindrical bore magnets performed better in the A‐P direction, with 1.4% and 12.6% of voxels respectively for the 1.5 T and 3.0 T, and with no voxels yielding distortions over 2 mm. The differences in the amount of distortion for the S‐I axis are less apparent, however the maximum distortion for the two cylindrical bore magnets is about half of those seen on the open‐bore magnet.

## 4 Discussion

This work sought to design, optimize, and build a modular 3D large FOV distortion phantom and implement residual GNL distortion characterization in a widely available software platform. This phantom features a modular design allowing for the flexibility to custom tailor the phantom shape in order to characterize many different MR and MR‐IGRT systems. Notably, the phantom could accommodate a FOV of 55 × 55 × 45 cm^3^ for the largest bore size we measured (70 cm). Early phantom designs, such as the phantom used by Breeuwer et al., focused on small regions of interest near isocenter, and thus did not characterize distortion at the periphery of the FOV^10^. Other phantoms built may not extend to cover the entire FOV needed to support MR‐only treatment planning of larger body sites such as the pelvis or for wide‐bore configurations^3^. Huang et al. limited their phantom build in the S‐I dimension to reduce the weight^11^, although the entire FOV could be sampled by stepping the phantom through various couch positions within the bore as described in our previous work^8^.

While the modular design implemented in this work offers flexibility to accommodate many different sized bores, the reassembly of the plates may cause differences in control point locations and could potentially lead to errors in control point locations. However, the eight threaded rods and tightened bolts help to stabilize the phantom for different setups. Further, any slight deviations in phantom reassembly could be addressed by acquiring an updated CT reference dataset for the exact phantom configuration that would be used at the time of MR acquisition. Overall, the modular phantom design was found to be stable upon repeated setups (setup uncertainty < 0.7 mm in all dimensions). Another limitation of our phantom design is the large weight which is consistent with other designs in the literature^11^, ^12^. While the phantom can be disassembled if desired for portability, the extended build utilizing all 20 plates weighed a total of ~44 kg and required two staff members to assist with phantom setup. Nevertheless, it is likely that after initial characterization, full FOV distortion measurements would not frequently be required as our previous work showed that the GNL distortion was stable over > 6 months of characterization. Recently, annual large FOV distortion measurements were recommended in the literature^25^. An alternative phantom build would be to use lighter density foam as done in previous iterations of this design; however, previous generations were also prone to damage, which can be problematic for maintaining the geometric integrity of the phantom. Another option would be to build the phantom with a shortened S‐I extent, similar to Huang et al., and to step the phantom to cover the entire FOV^11^.

A significant challenge when building the phantom was the time required to precision‐machine the polyurethane foam to accommodate ~7,500 paintball landmarks. Other prototypes of similar phantoms contained a more variable density sampling pattern that would reduce the amount of machining and paintballs required, with decreased landmarks near the center of the phantom where distortions are minimal and increased number of landmarks near the periphery where more fine sampling is needed^13^, ^26^. Our modular phantom design provides the option of filling only some of the control points with paintballs as needed. Because the phantom required variable plate widths to accommodate the tapered design, the machining template required multiple modifications during the phantom generation. In addition, the thickness tolerance of the polyurethane foam plates was quite variable requiring additional machining to bring the plate thickness to the specified tolerance. Finally, the paintballs rest inside the drilled holes without any affixing glue, and while they are flush with each plate surface, they often became dislodged and required reseating when phantom configuration changes are made. An alternative solution to the heavy design and inclusion of many landmarks has been proposed by Tadic et al., who uses a harmonic approach using a limited set of measurements of the distortion at the boundary of a phantom or region of interest^27^, ^28^ which is currently under commercial development.

The software validation shows nearly equivalent results for distortion in all axes between the old methods (stepped phantom with MATLAB software) and the new methods (large modular phantom with C++ software). The new methods measure distortions of less than 1 mm up to about 10 cm radial distance from isocenter in both the L‐R and A‐P directions, with distortion increasing nonlinearly as radius increases, which are consistent with previous results^8^. The S‐I direction, however, showed a slightly higher magnitude of distortion and a wider distribution for the new phantom and software. A likely explanation includes the use of the CT reference dataset for assessment, which is also limited by its own inherent resolution (1.2 mm in‐plane resolution and 2 mm slice thickness used in this study). The new modular phantom also has a lower resolution of landmark placement than the previous phantom (25 mm vs 16 mm, respectively) although with a much larger extent (10 cm greater width and 20 cm greater height with the full build). The larger extent enables a better characterization of the edges of the FOV, where patients with large body habitus are likely to occupy. An advantage of the new phantom design is that one measurement will encompass the entire FOV, whereas in our previous analysis, a batch script file translated a phantom in the S‐I direction at three couch positions, possibly introducing additional uncertainty into the measurement process.

As was suggested by Wang et al., the multimagnet distortion characterization demonstrated significantly worse distortions for the open‐bore 1.0 T MRI than for either cylindrical bore magnet^29^. However, even though all images were taken with vendor‐provided 3D distortion corrections enabled, all three MR systems yielded distortions over 1 mm at radii greater than 10 cm for at least two axes. These measurements are consistent with a recent study comparing the overall distortions for multiple magnets and vendors^24^. Also, for both our study and Walker et al.*,* the remaining distortion postcorrection for the cylindrical bore magnets increases gradually with increasing radius, with maximum distortions (near 20 cm from isocenter) of 2–3 mm^24^. It is important to note that the measurements obtained in our work were obtained with the image shutter, characterized as the centermost 45 cm FOV, turned off. Thus, we characterized data outside of this region of interest that is not recommended to be used by the vendor. The distortions in the A‐P axis were much smaller for cylindrical bore magnets, and, for the 1.5 T Ingenia, were smaller than 1 mm for nearly the entire FOV. Additionally, increased distortion in the through‐plane direction (S‐I) for cylindrical bore magnets has also reported in a recent study by Torfeh et al.^30^. Here, except near isocenter, the authors found that the through‐plane (S‐I) distortion was consistently higher than the in‐plane distortion for both 2D and 3D sequences. Possible causes include the gradient design for this axis or shimming in the S‐I dimension. It is also worth noting that the data shown in Fig. 6 for the Panorama do not cover as large of a radius as the other bores due to the smaller useable FOV of the open‐bore design in the S‐I direction. Future work will evaluate the GNL for other manufacturers, including additional magnet configurations for MR‐IGRT.

While the current version of the software developed for this study is limited to automated distortion characterization for our phantom design, it creates necessary tools for semi‐automated distortion characterization on other phantoms utilizing point‐like landmarks, allowing for potential widespread implementation into the community. However, before the module is made publically available, it is important to first implement a robust verification and validation of the code for different hardware and software configurations. It is the goal of the authors to use an approach similar to that described in a previous study by Pinter et al.*,* which developed an extensive RT toolkit for 3D Slicer that was made widely available to the RT community^31^. Notable validation steps were performed including using the CTest test system to perform nightly tests using reference input data and automatically comparing these results to a baseline solution. Future work will also include developing and implementing modules for synCT generation and patient‐specific distortion into the same 3D Slicer toolkit to support an MR‐only treatment planning workflow.

## 5 Conclusion

We optimized the design and implementation of a modular, extendable distortion phantom to support an MR‐only workflow and MR‐IGRT. A modular phantom design was deemed necessary for large FOV distortion characterization to accommodate a wide range of bore sizes and configurations. Utility was shown for three different bore designs. The phantom blueprints and accompanying analysis software will be widely available through online libraries, which will help to facilitate collaboration and multi‐institutional trials for MR‐only treatment planning.
